# Resistome Signature and Antibiotic Resistance Mechanisms in Rhizospheric Soil Bacteriomes of Mecca Region, Saudi Arabia: Insights into Impact on Human Health

**DOI:** 10.3390/life14080928

**Published:** 2024-07-24

**Authors:** Rewaa S. Jalal, Hana S. Sonbol

**Affiliations:** 1 Department of Biological Sciences, College of Science, University of Jeddah, Jeddah 21493, Saudi Arabia; rsjalal@uj.edu.sa; 2 Department of Biology, College of Science, Princess Nourah bint Abdulrahman University, Riyadh 11671, Saudi Arabia

**Keywords:** ARG, opportunistic, HGT, prevalent, *Pseudomonas aeruginosa*, *Mycobacterium tuberculosis*

## Abstract

The objective of this investigation is to ascertain the distinctive profile of the rhizospheric soil resistome within the Mecca region, while also evaluating the potential risks associated with the horizontal transfer of resistome determinants to the open environment and human clinical isolates. We have made metagenomic whole-genome shotgun sequencing for rhizospheric microbiomes of two endemic plants, namely *Moringa oleifera* and *Abutilon fruticosum*. The rhizospheric resistomes of the two plants and the abundance of antibiotic resistance genes (ARGs) were identified by cross-referencing encoded proteins with the comprehensive antibiotic resistance database (CARD). The identified ARGs were then analyzed for their antimicrobial resistance (AMR) mechanisms. Predominantly within this soil are the two bacterial species *Pseudomonas aeruginosa* and *Mycobacterium tuberculosis*. These opportunistic human pathogens are implicated in respiratory infections and are correlated with heightened mortality rates. The most prevalent array of ARGs existing in this soil comprises *mexA*, *mexC*, *mexE*, and *cpxR*, associated with mechanisms of antibiotic active efflux, along with *ACC*(2), *ACC*(3), *AAC*(6), and *APH*(6), in addition to *arr1*, *arr3*, *arr4*, *iri*, *rphA*, and *rphB*, implicated in antibiotic inactivation. Furthermore, *vanS*, *vanR*, and *vanJ* are identified for antibiotic target alteration, while *rpoB2* and *RbpA* are noted for antibiotic target replacement and protection, respectively. These mechanisms confer resistance against a diverse spectrum of drug classes encompassing fluoroquinolones, aminoglycosides, glycopeptides, and rifampicins. This study underscores the potential hazards posed to human health by the presence of these pathogenic bacteria within the rhizospheric soil of the Mecca region, particularly in scenarios where novel ARGs prevalent in human populations are harbored and subsequently transmitted through the food chain to human clinical isolates. Consequently, stringent adherence to good agricultural and food transportation practices is imperative, particularly with regard to edible plant parts and those utilized in folkloric medicine.

## 1. Introduction

Local communities possess critical indigenous knowledge essential for the sustainable management of natural resources, including wild plant species [1]. Within this context, *Moringa oleifera* and *Abutilon fruticosum*, which belong to the Moringaceae and Malvaceae families, respectively, are notable for their exceptional resilience in the arid climates of Saudi Arabia [2,3]. *M. oleifera* stands out for its diverse economic potential, spanning nutrition, traditional medicine, industry, pharmaceuticals, and agriculture [4]. Abundant in essential vitamins, minerals, and antioxidants, *M. oleifera* synthesizes compounds beneficial for blood pressure and cholesterol regulation, with established traditional medicinal uses against chronic diseases and liver ailments [5,6]. Its versatility extends to water purification and the biosynthesis of varied products, with recent research highlighting the stability and dermatological advantages of its seed oil [7]. *A. fruticosum* has also garnered notable scientific interest for its therapeutic and pharmacological attributes [8]. Scholarly discourse underscores the biochemical intricacies within *A. fruticosum*, emphasizing vital metabolites predominantly found in its foliage, seeds, and flowers Suryawanshi [9]. Empirical findings substantiate its potent antibacterial efficacy against respiratory pathogens, like *E. coli* and *P. aeruginosa*, alongside its cultural reverence as a natural remedy for diverse maladies [10]. Together, the medicinal and cultural significance of these two wild plant species underscores their profound value in traditional and folkloric practices [8,11,12].

The advent of high-throughput sequencing technologies has revolutionized the understanding of microbial communities in the rhizospheric environment. Amplicon and metagenomic whole-genome shotgun sequencing (mWGS) have emerged as pivotal methodologies for dissecting the intricacies of rhizospheric bacterial consortia [13,14]. While databases, like SILVA and Greengenes, have facilitated taxonomic characterization, 16S rRNA sequencing faces limitations in identifying viral entities [15,16]. On the other hand, mWGS provides precise identification of microbial genomes and offers detailed insights into gene abundance and metabolic dynamics across different ecological niches [17,18]. It also allows the examination of genes encoding the resistome [19], as well as CAZymes (carbohydrate-active enzymes) to be involved in cross-talking functional pathways [20].

The resistome refers to the assortment of antibiotic resistance genes (ARGs) harbored in the microbiome of a specific ecological niche [21]. Building on recent advances in resistome research, future directions have been proposed to enhance our understanding and management of ARG transmission [22]. Furthermore, upcoming climate scenarios exacerbate health risks associated with the soil microbiome by reshaping both the resistome and pathogenome [23,24]. Advanced research methodologies are crucial for unraveling the intricate structure of the resistome within environmental microbiomes. Within such environments, newly emerging bacterial genotypes may acquire the capacity to synthesize novel ARGs and transfer them horizontally via their mobile genetic elements (MGEs) to closely related human pathogens [25]. The risk of horizontal gene transfer (HGT) intensifies in regions of rhizospheric soil where pathogenic bacteria harbor ARGs prevalent in the human population, particularly when the rhizosphere supports the growth of edible plants for human or livestock consumption, or plants with commercial importance [26]. Noteworthy examples of these antibiotic-resistant “superbugs” encompass genera such as *Pseudomonas*, *Mycobacterium*, and *Staphylococcus* [27]. A noteworthy instance entails the transfer of the *ampC* gene conferring resistance to beta-lactam antibiotics to the opportunistic human pathogen *Pseudomonas aeruginosa* [28].

The main target of the present study was to explore resistome and their resistance mechanisms in two locations of the bacterial rhizospheric soil microbiomes in the Macca region, Saudi Arabia. As a case study, we will focus on the rhizospheric soil of two wild plants, *Moringa oleifera* and *Abutilon fruticosum*, native to this region. Initial analysis revealed 51 ARGs commonly present in bacteriomes of both plant rhizospheres and contribute to five antibiotic resistance mechanisms. We will also assess the prevalence of the bacteria carrying these ARGs in rhizospheric soils of these plants, as some bacteria are recognized as human pathogens that could pose a direct risk if their ARGs are horizontally transferred to clinical strains of closely related species.

## 2. Materials and Methods

### 2.1. Sample Collection and DNA Extraction

Six rhizospheric soil samples (50 g) were collected in Winter 2021 from naturally existing *Moringa oleifera* and *Abutilon fruticosum* wild plants in the northwestern region of the Mecca region, Saudi Arabia, situated close to the coast of the Red Sea at coordinates 21.209430/39.530866 and 21.352751/39.578932, respectively [29]. The Mecca region was selected due to its status as the area with the highest diversity of medicinal plants in Saudi Arabia. The plant species chosen were picked for their roles in the food chain and their significance across agriculture, pharmaceuticals, and medicine. Additionally, these species are among the most commonly found species throughout the Mecca region. The chosen area for experimentation had experienced no precipitation for over 3 months prior to sampling, and the selected plants were individually grown, displaying uniform growth characteristics. Lateral roots were cut at depths between 10 and 30 cm to collect the microbiomes from the rhizosphere. Subsequently, soil that was not physically attached to the root but located within 1 cm of the root was gathered [30]. Simultaneously, three bulk soil samples were obtained at the same depth from locations approximately 10 m apart from *M. oleifera* and *A. fruticosum* plants selected for the experiment. Upon collection, samples were preserved at −20 °C. DNA from various specimens was extracted utilizing CTAB/SDS, and concentration was adjusted to 10 ng/μL, following established protocols [31].

### 2.2. Whole-Genome Shotgun Sequencing and Bioinformatics Analysis

An amount of 30 μL of each DNA sample was dispatched to Novogene Co. (Helios, Singapore) to perform comprehensive whole-metagenome sequencing. The raw data derived from the microbiomes of the two indigenous plant species were archived in the European Nucleotide Archive (ENA). Accession numbers ERR10100770-72 were assigned to rhizospheric samples, while ERR10100773-74 and ERR10100781 were designated for bulk soil samples of *M. oleifera*. For *A. fruticosum*, accession numbers ERS15580318-ERS15580320 and ERS15580321–ERS15580323 for bulk and rhizospheric soils of the two plants were, respectively, assigned. Stringent quality control measures were applied, where the initial processing of raw sequencing data required eliminating bases with quality scores ≤ 38 that extended beyond a 40-base-pair threshold and removing reads containing 10 or more ambiguous base pairs (Ns). Library preparation ensued and was followed by sequencing of clean data as previously described [31]. The assembled data underwent rigorous processing, with the elimination of chimeras as per established protocols [32]. Detailed downstream analyses were performed as previously described [33,34]. Software used in analyzing the raw sequencing data include MEGAHIT v.1.2.9 for *de novo* assembly [33], SOAP v2.21 for comprehensive mapping (https://GitHub.com/ShujiaHuang/SOAPaligner, accessed on 5 May 2024), MetaGeneMark-2 for annotation of highly abundant genes [35], Cluster Database at High Identity with Tolerance (CD-HIT) v.4.8.1 for subsequent gene set replication [36], MEGAN v6 binning reference-based classification algorithm for functional analysis [37], and DIAMOND v2.1.8 for mapping-deduced amino acid sequences [38].

To elucidate the resistome, encoded proteins were cross-referenced against the CARD, enabling the recovery and assessment of ARGs for abundance [39,40]. These genes were subsequently searched for antimicrobial resistance (AMR) mechanisms following established methodologies [41]. Subsequently, chosen antibiotic resistance gene (ARG) families or groups underwent analysis to identify the resistome signature in the Mecca region.

## 3. Results

**Figure 1:**
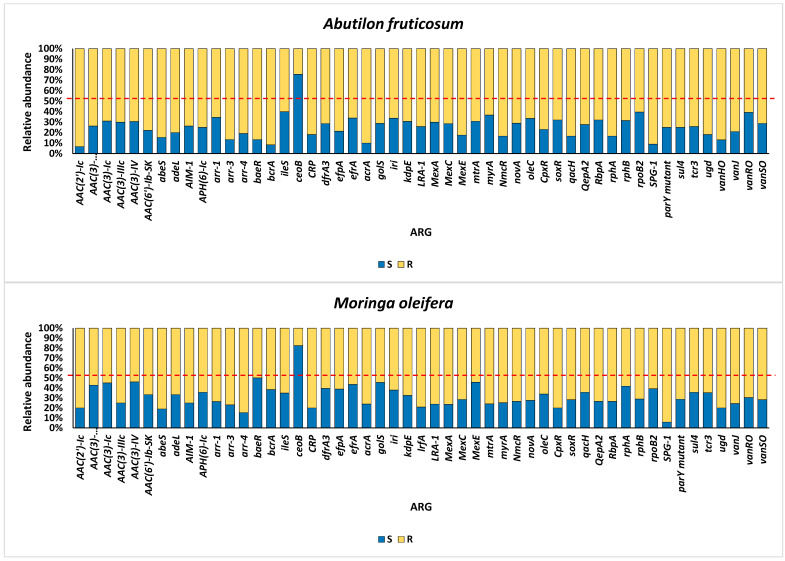
Relative abundance of ARGs frequently encountered concurrently within the rhizospheric (R) and bulk (S) soil microbiomes of the two wild botanical specimens *Moringa oleifera* and *Abutilon fruticosum*. The red line represents a 50% relative abundance. Further information is available in Tables S3 and S4.

**Figure 2:**
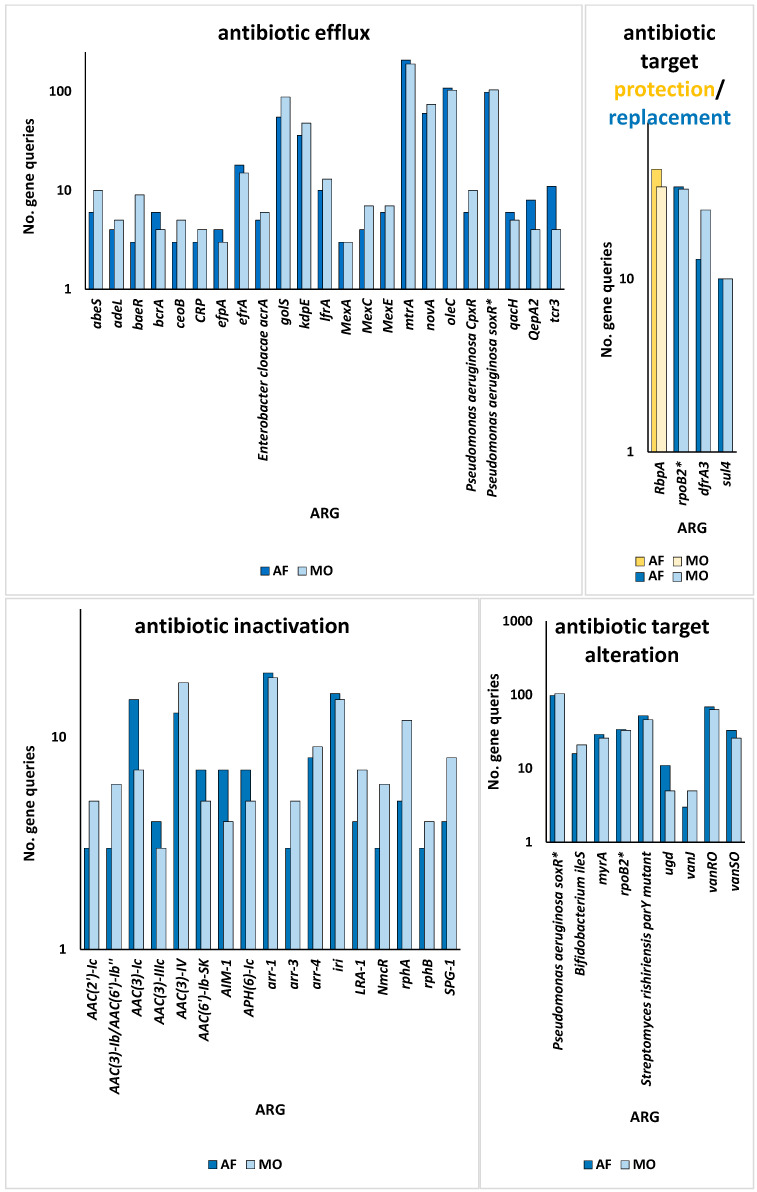
Number of non-redundant queries pertaining to antibiotic resistance genes (ARGs), inclusive of their associated resistance mechanisms, prevalent within the rhizospheric soil microbiomes of *Moringa oleifera* (MO) and *Abutilon fruticosum* (AF). Asterisks (*) refer to ARGs with more than one resistance mechanism. Further information is available in Tables S5–S10.

**Figure 3:**
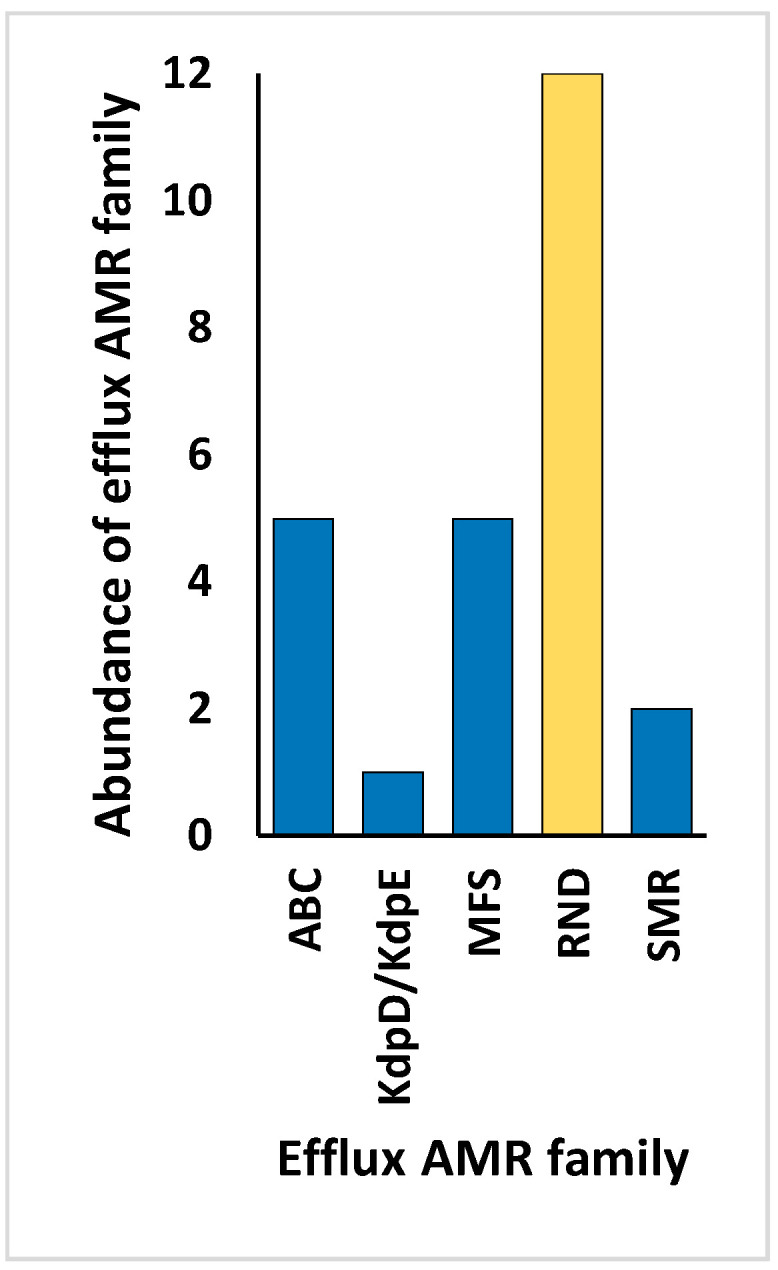
Abundance of efflux antimicrobial resistance (AMR) families within the rhizospheric soil microbiomes of the two indigenous botanical taxa *Moringa oleifera* and *Abutilon fruticosum*. The column highlighted in orange corresponds to the dominant AMR family housing the most widespread antibiotic resistance genes (ARGs). Further information is available in Tables S5 and S6.

**Figure 4:**
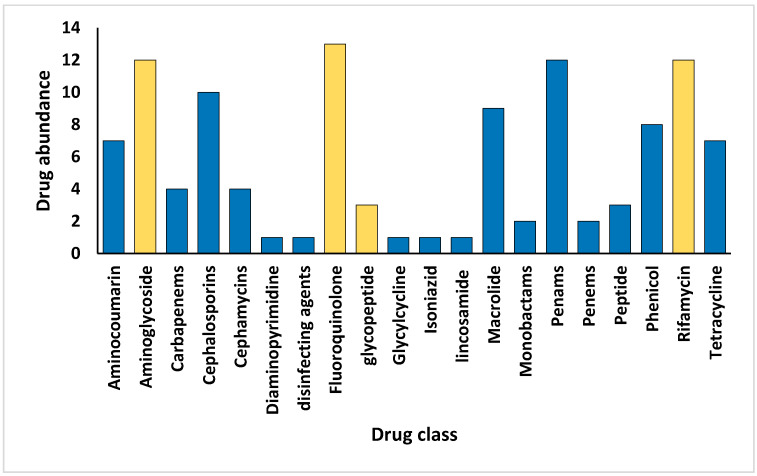
Abundance or number of antibiotics within drug classes targeted by ARGs prevalent within the rhizospheric soil microbiomes of *Moringa oleifera* and *Abutilon fruticosum*, underscoring a nuanced interplay between microbial ecology and pharmaceutical dynamics. The columns highlighted in orange show a range of drug types that are at risk from the wide variety of ARG families present in the rhizospheric environment of these wild plant species. This helps to reveal the complex ways in which resistance spreads in these ecological niches and provides insights for further analysis. Further information is available in Tables S6–S10.

Aligned with the primary aim of this study, we examined the resistomes of the rhizospheric soil of the two indigenous plants, *Moringa oleifera* and *Abutilon fruticosum,* across two locations within the Mecca region. Our investigation focused on the elucidation of the ARGs and associated resistance mechanisms inherent within the microbiomes of this geographic area. The findings outlined in Tables S1 and S2 reveal the presence of 183 ARGs within the soil of *M. oleifera*, with a range of gene queries spanning from 1 to 189. Similarly, the rhizospheric soil of *A. fruticosum* harbored 154 ARGs, with gene queries ranging from 1 to 208. Upon comparison, 51 ARGs were found to be shared between these two environments, excluding ARGs with ≤2 queries. The abundance of these antibiotic resistance genes (ARGs) generally demonstrated elevated occurrences within the rhizospheric soil of both wild plant species in contrast to the levels observed in the bulk soil (Tables S3 and S4). Accordingly, the relative abundance of these ARGs tended to be greater within the microbiomes of rhizospheric soil than those of the bulk soil, with the exception of the *ceoB* gene, which displayed contrasting results (Figure 1). These ARGs operate through five distinct resistance mechanisms, including antibiotic active efflux, antibiotic inactivation, antibiotic target alteration, antibiotic target replacement, and antibiotic target protection (Figure 2 and Table S5). The attributes of these ARGs, encompassing their AMR family, drug class, and the genus hosting these ARGs for each of the five resistance mechanisms, are delineated in Tables S6–S10, utilizing data extracted from the CARD (https://card.mcmaster.ca, accessed on 5 May 2024). Notably, *soxR* and *rpoB2* genes are involved in multiple resistance mechanisms, with the former contributing to antibiotic efflux and target alteration, and the latter to antibiotic target alteration and replacement (Table S5). Among these mechanisms, the family of the resistance-nodulation-cell division (RND) efflux pump has emerged as the most prevalent AMR family within the rhizospheric soil microbiomes of both plant species (Figure 3). Regarding the targeted drug classes of the identified ARGs, aminoglycosides, fluoroquinolones, penams, and rifamycins were the most frequently encountered within the rhizospheric soil microbiomes of *M. oleifera* and *A. fruticosum* (Figure 4). However, glycopeptides appear to be a prime focus of a significant ARG family found in the complex *vanRSJKHAX* operon, which plays a role in altering the target of vancomycin antibiotics. Consequently, we opted to examine the ARGs within this operon that are common in the resistomes of the two wild plants.

**Figure 5:**
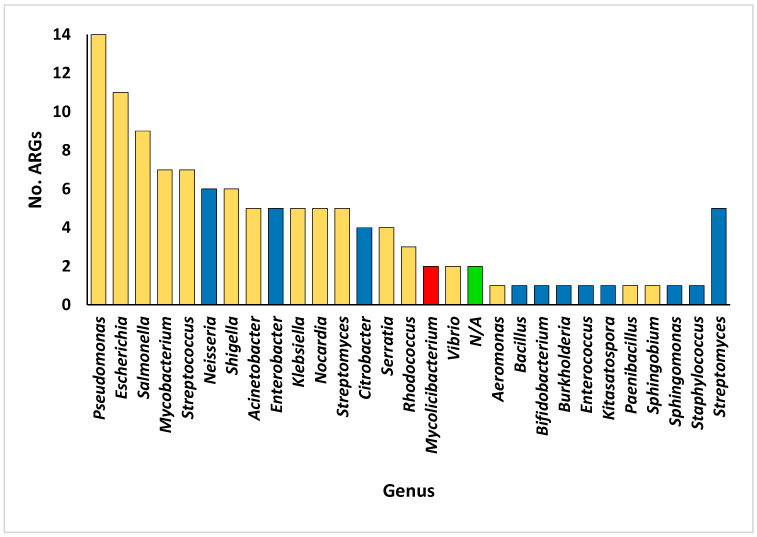
Number of antibiotic resistance genes (ARGs) within the bacterial genera present in the rhizospheric soil microbiomes of *Moringa oleifera* and *Abutilon fruticosum*. Noteworthily, the orange columns delineate bacterial taxa boasting a rich reservoir of diverse ARG families, elucidating the multifaceted nature of resistance potential within these ecological niches. Conversely, the green column (N/A) elucidates a distinct scenario, highlighting the presence of two unique ARGs, *novA* and *LRA-1*, which transcend the confinement to specific bacterial genera, thus accentuating their broader distribution and evolutionary significance within these plant-associated microbial communities. The red column indicates a bacterial genus not present in the rhizospheric soil of either plant species, which was not subjected to further analysis. Further information is available in Tables S6–S10.

**Figure 6:**
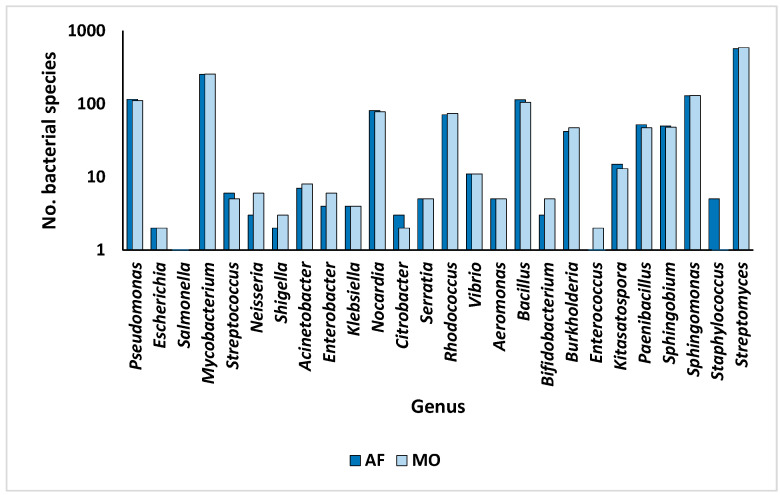
Number of species of the bacterial genera hosting antibiotic resistance genes (ARGs) within the rhizospheric soil microbiomes of *Moringa oleifera* (MO) and *Abutilon fruticosum* (AF). Further information is available in Tables S14 and S15.

**Figure 7:**
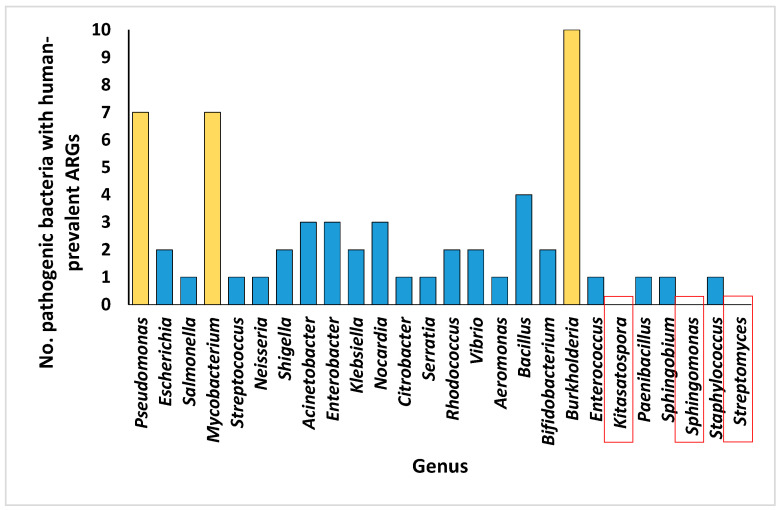
Number of pathogenic bacteria with human-prevalent ARGs within the rhizospheric soil microbiomes of *Moringa oleifera* (MO) and *Abutilon fruticosum* (AF). Orange columns denote the predominant bacterial genera identified as human pathogens in rhizospheric soils of the two plant species. Highlighted in red squares are bacterial genera devoid of human pathogenic species. Further information is available in Tables S11–S15.

The number of ARGs at the bacterial genus level varies from one to fourteen within the rhizospheric soil microbiomes of the two plant species (Figure 5 and Tables S6–S10). Notably, *Bacillus*, *Bifidobacterium*, *Burkholderia*, *Enterococcus*, *Staphylococcus*, and *Streptomyces* exhibited the lowest number of ARGs, while *Pseudomonas* emerged as the genus with the highest abundance of ARGs, followed by *Escherichia*, *Salmonella*, *Mycobacterium*, and *Streptococcus*. Furthermore, the examination of pathogenic bacterial inhabitants housing prevalent ARGs in humans revealed 435 instances (Table S11). Of these species, 59 were recognized as pathogenic bacterial residents within the rhizospheric soil microbiomes of the two plants with prevalent ARGs in humans. The distribution of all bacterial genera/species within the rhizospheric soil microbiomes of the two plants is delineated in Tables S12 and S13, with specific focus on genera hosting ARGs in microbiomes of the rhizospheric soil of the two plants illustrated in Tables S14 and S15. The number of bacterial species associated with the genera hosting ARGs is depicted in Figure 6. Notably, *Streptomyces* exhibited the highest species diversity, followed by *Mycobacterium*, *Sphingomonas*, *Pseudomonas*, and *Bacillus* (Tables S14 and S15). Notable genera harboring significant pathogenic species hosting prevalent ARGs in humans include *Pseudomonas*, *Burkholderia*, and *Mycobacterium*, whereas bacterial genera lacking human pathogenic species comprise *Kitasatospora*, *Sphingomonas*, and *Streptomyces* (Figure 7).

**Figure 8:**
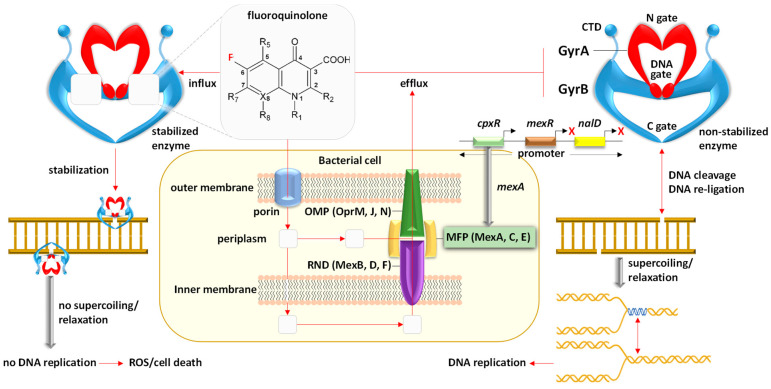
Participation of the antibiotic resistance genes (ARGs) *mexA*, *mexC*, *mexE*, and *cpxR* in the operation of RND multidrug tripartite membrane-bound efflux pumps MexAB–OprM, MexCD–OprJ, and MexEF–OprN in the Gram-negative bacteria *Pseudomonas aeruginosa*. The ARG cpxR facilitates augmented expression of the efflux pump MexAB-OprM. These complex interactions culminate in the expulsion of fluoroquinolone antibiotics, essential for gyrase enzyme stabilization and consequent inhibition of bacterial DNA replication and cell death. Green boxes denote predominant proteins/genes within the rhizospheric soil microbiomes of *Moringa oleifera* and *Abutilon fruticosum*. OMP = outer membrane protein, MFP = membrane fusion protein, RND = resistance nodulation division. GyrA and GyrB constitute integral components of the gyrase enzyme. Further information is available in Table S6.

**Figure 9:**
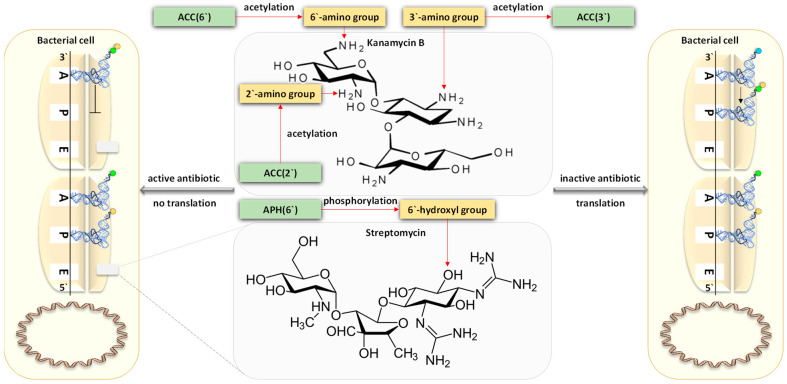
Engagement of antibiotic resistance genes (ARGs) *ACC(2*′*)*, *ACC(3*′*)*, and *ACC(6*′*)* in the acetylation process targeting the 2′, 3′, and 6′ amino groups of kanamycin B, alongside the ARG *APH(6*′*)* in the phosphorylation of streptomycin, resulting in the inactivation of these two aminoglycoside antibiotics in the Gram-positive bacteria *Mycobacterium tuberculosis* and the Gram-negative bacteria *Pseudomonas aeruginosa*. The mechanism of action of aminoglycoside antibiotics involves binding to the 30S ribosomal subunit of the 70S ribosomes within bacterial cells, inhibiting the translocation of peptidyl-tRNA from the A-site to the P-site. This interference hampers bacterial growth by disrupting protein translation processes. Green boxes denote predominant proteins within the rhizospheric soil microbiomes of *Moringa oleifera* and *Abutilon fruticosum*. Further information is available in Table S7.

**Figure 10:**
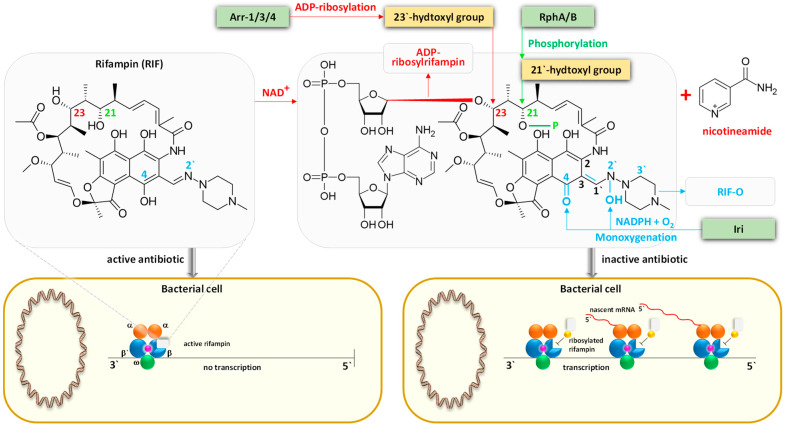
The involvement of antibiotic resistance genes (ARGs) *arr1*, *arr3*, and *arr4* in the ADP-ribosylation cascade targeting the hydroxyl group of carbon atom 23 within rifampin (RIF) antibiotics, shown concurrently with the participation of ARGs *rphA* and *rphB* in the phosphorylation of the hydroxyl group of carbon atom 21, and *iri* in the monoxygenation, leading to antibiotic inactivation in the Gram-positive bacteria *Mycobacterium tuberculosis* and *Rhodococcus equi* and/or the Gram-negative bacteria *Pseudomonas aeruginosa*. The mechanism by which rifampins exert their action entails binding to the β subunit of the RNA polymerase enzyme, thereby obstructing the oligonucleotide exit tunnel and inhibiting the elongation of nascent mRNA strands. This obstruction thwarts bacterial proliferation by interfering with RNA transcription processes. Green boxes denote predominant proteins within the rhizospheric soil microbiomes of *Moringa oleifera* and *Abutilon fruticosum*. RIF-O = 2′-*N*-hydroxy-4-oxo-rifampcin. Further information is available in Table S7.

**Figure 11:**
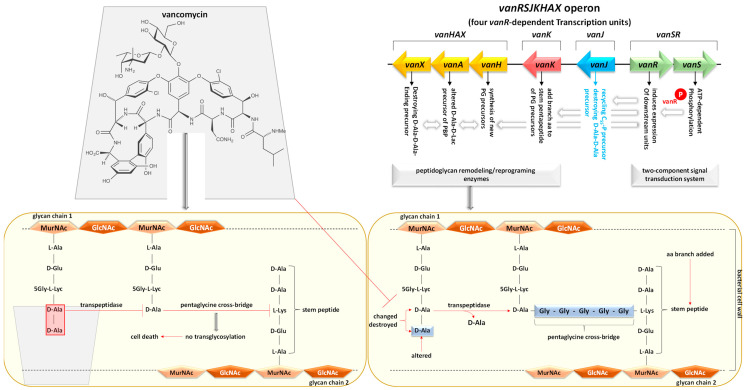
The involvement of antibiotic resistance genes (ARGs) *vanS*, *vanR*, and *vanJ* within the *vanRSJKHAX* operon, encompassing the four *vanR*-dependent transcription units that modulate the alteration of terminal D-Ala-D-Ala peptidoglycan precursors located in glycan chain 1 of cell walls of various Gram-positive bacteria targeted by the glycopeptide vancomycin antibiotics. The *vanS*, and *vanR* genes were discovered in the Gram-positive bacterial species *Rhodococcus equi* and *Nocardia* sp., while vanJ gene was discovered in the Gram-positive bacterium *Streptomyces coelicolor*. Vancomycin exert their mode of action by binding to the terminal D-Ala-D-Ala peptidoglycan precursors in the developing bacterial cell wall, inhibiting transglycosylation and inducing bacterial cell death due to the impeded pentaglycine cross-bridging with the stem peptide of glycan chain 2 of the bacterial cell wall. Green and blue arrows indicate prevalent genes within the rhizospheric soil microbiomes of *Moringa oleifera* and *Abutilon fruticosum*. Ala = alanine, aa = amino acid, PG = peptidoglycan. Further information is available in Table S8.

**Figure 12:**
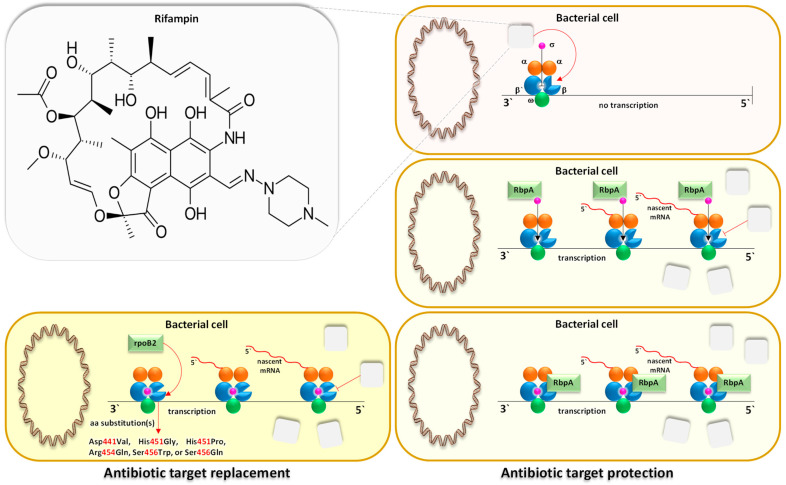
The participation of antibiotic resistance genes (ARGs) *rpoB2* and *RbpA* in the mechanisms of antibiotic target replacement and protection, respectively. *rpoB2* gene orchestrates amino acid substitutions within the β subunit of bacterial RNA polymerase (RNAP), specifically at positions Asp441Val, His451Gly, His451Pro, Arg454Gln, Ser456Trp, and/or Ser456Gln, disrupting rifampin’s binding affinity to RNAP in species of different genera including *Mycobacterium, Nocardia*, and/or *Rhodococcus*. Meanwhile, *RbpA* gene operates through distinct mechanisms to confer rifampin resistance. Firstly, it interacts with the σ factor of bacterial RNAP, inducing allosteric shift in the rifampin binding site. Secondly, it competes with rifampicin for the β binding site of RNAP. These concerted actions ultimately lead to diminished rifampicin affinity for RNAP, interfering with its canonical inhibition of the β subunit. Green boxes indicate prevalent ARGs within the rhizospheric soil microbiomes of *Moringa oleifera* and *Abutilon fruticosum*. The pink-shaded bacterial cells represent antibiotic-sensitive strains, whereas those shaded in beige and yellow denote antibiotic-resistant strains employing mechanisms of antibiotic target protection and antibiotic target replacement, respectively. Further information is available in Tables S9 and S10.

Utilizing the aforementioned findings, we identified the most prevalent gene families associated with the five resistance mechanisms and evaluated their respective target antibiotics, with a primary focus on the drug classes fluoroquinolones, aminoglycosides, glycopeptides, and rifamycins (Figure 8, Figure 9, Figure 10, Figure 11 and Figure 12). Fluoroquinolones encountered the ARGs primarily employing active antibiotic efflux (Figure 8), while aminoglycosides were challenged by the ARGs employing antibiotic inactivation (Figure 9), and glycopeptides were challenged by the ARGs employing antibiotic target alteration (Figure 11). For rifamycins, ARGs were found to employ mechanisms of antibiotic inactivation (Figure 10), antibiotic target replacement, and target protection (Figure 12).

ARGs involved in active antibiotic efflux include *mexA*, *mexC*, *mexE*, and *cpxR*, targeting fluoroquinolones (Figure 8), whereas those involved in antibiotic inactivation include *ACC(2)*, *ACC(3)*, *AAC(6)*, and *APH(6)*, targeting aminoglycosides (Figure 9), and *arr1*, *arr3*, *arr4*, *rphA*, *rphB*, and *iri* targeting rifamycins (Figure 10). ARGs involved in antibiotic target alteration include *vanS*, *vanR*, and *vanJ*, targeting glycopeptides (Figure 11), while those involved in antibiotic target replacement and protection include *rpoB2* and *RbpA*, respectively, targeting rifamycins (Figure 12). The RND multidrug tripartite membrane-bound efflux pumps MexAB–OprM, MexCD–OprJ, and MexEF–OprN were identified as the primary efflux antimicrobial resistance (AMR) family detected in this study (Figure 3 and Figure 8). The main functions employed by ARGs for antibiotic inactivation include acetylation, phosphorylation, ADP-ribosylation, and monoxygenation (Figure 9 and Figure 10), effectively preventing antibiotic binding to its target. ARGs altering antibiotic targets primarily alter terminal D-Ala-D-Ala peptidoglycan precursors, facilitating normal biosynthesis of the bacterial cell wall (Figure 11). Moreover, the ARG involved in the mechanism of antibiotic target replacement, exemplified by *rpoB2*, orchestrates the substitution of specific amino acid residues (such as Asp441Val, His451Gly, His451Pro, Arg454Gln, Ser456Trp, and/or Ser456Gln), thus eliciting a perturbation in the antibiotic binding site via allosteric modulation, as depicted in Figure 12. Furthermore, the protein product encoded by ARG is implicated in safeguarding antibiotic targets, engages in competitive interactions with antibiotics, and vies for occupancy at the binding locale of the β subunit of bacterial RNA polymerase (RNAP), as illustrated in Figure 12.

## 4. Discussion

One frequently employed therapeutic strategy for addressing bacterial infections across diverse contexts, encompassing human health, involves the utilization of antibiotics [42]. Antimicrobial resistance (AMR) has emerged as a prominent global apprehension, attributable to the escalating prevalence of drug resistance observed among bacterial strains. AMR represents a significant global health predicament, precipitating human mortality, as evidenced by a multitude of scholarly studies [43,44]. In the year 2019 alone, the toll of AMR-related fatalities surged to approximately 5 million individuals worldwide [45]. This information is publicly available in the Comprehensive Antibiotic Resistance Database (CARD). However, new databases have emerged to offer insights into the abundance and dynamics of environmental antibiotic resistance genes. These data are valuable for assessing health risks related to AMR [46].

### 4.1. Major Mechanisms of AMR in Rhizospheric Soil of the Two Wild Plants

In earlier reports, three fundamental mechanisms of AMR were suggested. They are enzymatic antibiotics degradation, alteration of bacterial proteins that are targets of antibiotics, and changes in membrane permeability that mediate a decrease in antibiotic influx and/or increase in antibiotic efflux [47]. These resistance mechanisms exhibited by antibiotic resistance genes (ARGs) typically impede the fundamental functions of antibiotics [42,48], which encompass the inhibition of key biological processes essential for cellular viability, namely DNA replication [49], RNA transcription [50,51], and protein translation [52], in addition to the inhibition of cell wall biosynthesis [53]. The identified ARG families in this study counteract these four modes of action of their respective targeted antibiotics (Figure 8, Figure 9, Figure 10, Figure 11 and Figure 12 and Table S5). Specifically, fluoroquinolones, rifamycins, and aminoglycosides inhibit bacterial DNA replication, RNA transcription, and protein translation, respectively, while glycopeptides inhibit cell wall biosynthesis (Figure 8, Figure 9, Figure 10, Figure 11 and Figure 12). The prevalent bacterial genera found to host ARGs within the soil rhizosphere of the two indigenous plant species engaged in the inhibition of these pharmaceutical agents and encompassed *Pseudomonas*, *Mycobacterium*, *Rhodococcus*, and *Nocardia*.

The average abundances of the predominant bacterial species were, respectively, 86.75, 1183.67, 421.08, and 62.92 for *M. oleifera*, and 152.62, 891.79, 314.03, and 66.63 for *A. fruticosum* (Tables S14 and S15). The numbers of the prevalent species within these genera were 7, 7, 2, and 3 for *M. oleifera*, and the corresponding values for *A. fruticosum*. Notably, the average abundance of the most harmful bacterial species, e.g., *Mycobacterium tuberculosis*, was as high as 5803.42 for *M. oleifera* and 4165.92 for *A. fruticosum*. Presently, there is a more comprehensive understanding of antibiotic resistance mechanisms in bacteria [44]. These mechanisms encompass active efflux, wherein transmembrane efflux pumps facilitate the expulsion of antibiotics from bacterial cells, consequently diminishing their intracellular concentration [54]. Conversely, the diminished influx is orchestrated by modifications in membrane permeability, achieved through the downregulation of porins. Porins, the integral transmembrane proteins pivotal in facilitating the translocation of a plethora of molecules, antibiotics among them, across bacterial membranes, undergo notable alterations [55].

Resistance mechanisms encompass the process of antibiotic inactivation, wherein specialized enzymes undertake the degradation or modification of antibiotics, consequently obstructing their ability to bind to their intended molecular targets [56]. Target site alteration entails structural changes in the antibiotic’s target to diminish its binding affinity [57]. Antibiotic target bypass occurs when the target’s action is fulfilled by a novel protein, which remains unaffected, rendering the target obsolete and the antibiotic ineffectual [58]. Typically, target protection entails the intricate interplay between a specific target protein and the antibiotic’s intended molecular target, effectively shielding the latter from inhibition induced by the antibiotic [59]. Antibiotic target replacement occurs when some critical amino acids in the antibiotic target site are substituted, resulting in a modified target and resistance to the respective antibiotics [51,60]. Subsequently, we demonstrate that five of these mechanisms of resistance have been detected in rhizospheric bacteria linked to the two plant species (Figure 8, Figure 9, Figure 10, Figure 11 and Figure 12 and Table S6).

#### 4.1.1. Active Antibiotic Efflux Pump

We have chosen to detect the involvement of specific ARGs including *mexA*, *mexC*, *mexE*, encoding integral components (e.g., membrane fusion proteins or MFP, Figure 8) of the RND tripartite membrane-bound efflux pumps MexAB–OprM, MexCD–OprJ, and MexEF–OprN, respectively [61]. In addition, the *cpxR* gene regulator was selected for its capacity to augment the expression of genes encoding the efflux pump MexAB-OprM [62], as depicted in Figure 8. These intricate molecular interactions orchestrate the active efflux of fluoroquinolones (FQs) antibiotics from the Gram-negative bacteria *Pseudomonas aeruginosa*.

The orchestration of regulatory mechanisms governing the functionality of the three efflux pumps is intricately tailored to the exigencies of their respective roles. For instance, the MexAB-OprM efflux system serves as a linchpin in the resistance repertoire of *P. aeruginosa* against quinolones and a plethora of other antibiotics [63]. The augmented expression of this system engenders a broad spectrum of cross-resistance to multiple antibiotic classes [64], while concurrently facilitating the extrusion of quorum-sensing (QS) mediators, thereby stimulating the synthesis of virulence factors [65]. Conversely, the regulatory dynamics governing the MexEF-OprN system unfold subsequently, concomitant with the waning of MexAB-OprM expression [66]. Notably, the downregulation of the *mexB* gene correlates with the upregulation of *mexE*, indicative of divergent regulatory paradigms governing these two efflux pumps [67]. In essence, the upregulation of MexAB-OprM engenders a concomitant attenuation of the QS response, whereas the heightened expression of MexEF-OprN and MexCD-OprJ culminates in a compromised QS response [68].

Fluoroquinolones (FQs) exert their antimicrobial activity primarily by targeting bacterial enzymes such as DNA gyrase, pivotal in DNA manipulation processes encompassing DNA breakage, the passage of duplex DNA through the break, and the subsequent resealing to facilitate DNA relaxation, replication, and cell division [69]. Mechanistically, FQs operate by forming a complex with DNA gyrase (Figure 8), thereby stabilizing the enzyme and impeding bacterial DNA relaxation and replication. These inhibitory actions culminate in the accumulation of reactive oxygen species and consequently cause bacterial cell death [70].

*P. aeruginosa* harbors 12 efflux pumps of the RND family of transporters, pivotal in both intrinsic and acquired multidrug resistance mechanisms among all Gram-negative bacteria [71]. This bacterial species is a prevalent nosocomial opportunistic human pathogen associated with elevated mortality rates that exhibits remarkable adaptability across diverse ecological niches, including soil habitats [63,68,72]. In the rhizobiome of both plant species, this bacterium exhibits a plethora of closely related pathogenic and non-pathogenic species as illustrated in Tables S14 and S15, which harbor an abundant array of ARGs, as illustrated in Figure 5, Figure 6 and Figure 7. Hence, it is plausible that horizontal gene transfer (HGT) transpires within the soil microbiomes of the two plant species.

#### 4.1.2. Antibiotic Inactivation

In delineating this resistance mechanism, we scrutinized two groups of ARGs. The first encompasses *ACC(2)*, *ACC(3)*, and *ACC(6)*, which encode acetyltransferases, targeting the acetylation of amino groups aminoglycoside antibiotics at positions 2′, 3′, and 6′, respectively, alongside the *APH(6*′*)* gene responsible for the phosphorylation of these antibiotics (Figure 9). The second group comprises *arr1*, *arr3*, and *arr4* encoding three types of rifampin ADP-ribosyltransferases (Figure 10). This group is implicated in the ADP-ribosylation of the hydroxyl group located at carbon atom 23 within rifampicin (RIF) antibiotics, concomitantly with ARGs *rphA* and *rphB*, involved in the phosphorylation of the hydroxyl group positioned at carbon atom 21 of RIF, and *iri*, involved in the monoxygenation/decolorization of the carbon atoms 2′ and 4 of RIF [73]. These two intricate molecular interactions precipitate the inactivation of these drug class across various Gram-positive and Gram-negative bacterial species, encompassing *Mycobacterium tuberculosis* and *Pseudomonas aeruginosa*, respectively [74].

The mode of action of aminoglycosides involves inhibiting protein translation as they bind to 30S ribosomal subunit of the 70S ribosomes within bacterial cells, inhibiting the translocation of peptidyl-tRNA from the A-site of the large ribosomal subunit to the P-site [75]. Conversely, the function of RIF involves binding to the beta subunit of RNAP, obstructing the oligonucleotide exit tunnel and impeding the elongation of nascent mRNA strands. This hindrance disrupts bacterial proliferation by interfering with RNA transcription [76,77].

*Mycobacterium tuberculosis*, the bacterium responsible for tuberculosis (TB), is a respiratory infection that presents a major global public health challenge [78], particularly due to its resistance to rifampicin (RIF) and isoniazid (INH), the primary first-line drugs against tuberculosis (TB) [51,79]. Treatment typically involves two phases: the initial (intensive) phase, which includes the first-line drugs, and the continuation phase, which also includes first-line drugs but often with a focus on INH and RIF due to resistance concerns. [80]. The standard treatment for active tuberculosis (TB) involves a combination of the two first-line drugs INH and RIF in addition to pyrazinamide (PZA) and ethambutol (EMB). Due to the significant risk posed by the resistance to isoniazid and rifampin, these drugs are continued into the continuation phase. This standardized treatment regimen aims to minimize the risk of developing further drug resistance and to ensure successful treatment outcomes for most patients. It is typically manifested in individuals with human immunodeficiency virus (HIV) [81]. As a multidrug-resistant (MDR) pathogen, *M. tuberculosis* also demonstrates resistance to the aminoglycosides kanamycin and amikacin, both crucial bactericidal agents utilized in MDR TB treatment regimens [82]. Notably, the resistance to either or both of these agents is indicative of extensively drug-resistant TB. Within the rhizobiome of both plant species, a diverse array of closely related pathogenic and non-pathogenic *M. tuberculosis* strains exists, as depicted in Tables S14 and S15. These bacteria harbor a multitude of ARGs, as depicted in Figure 5, Figure 6 and Figure 7. Therefore, the prospect of horizontal gene transfer (HGT) taking place within the microbiomes of the rhizospheric soils associated with these two plant species is substantial.

#### 4.1.3. Antibiotic Target Alteration

For this particular mode of resistance, we have discerned the presence of the ARGs *vanS*, *vanR*, and *vanJ*, all situated within the *vanRSJKHAX* operon (Figure 11). Among these ARGs, *vanS* and *vanR* are pivotal in Gram-positive bacterial species like *Rhodococcus equi* and *Nocardia* sp. in conferring resistance against glycopeptide antibiotics such as vancomycin and teicoplanin [83]. Conversely, *vanJ* represents a novel membrane protein instrumental in bestowing resistance to teicoplanin and its derivatives in the Gram-positive bacterium *Streptomyces coelicolor*. The function of *vanJ* involves the recycling of undecaprenol pyrophosphate during the biosynthesis of cell wall components [84]. This operon comprises four vanR-dependent transcription units, coordinating intricate processes such as peptidoglycan remodeling/reprogramming and culminating in alterations to the terminal D-Ala-D-Ala peptidoglycan precursors situated within glycan chain 1 of bacterial cell walls. Additionally, it governs transpeptidase activity and facilitates pentaglycine cross-bridging to link the stem peptide of glycan chain 2 [83,85].

Glycopeptide antibiotics, synthesized non-ribosomally by Actinomycetales, function by binding to the D-Ala-D-Ala terminal peptidoglycan precursors within the nascent bacterial cell wall. This binding event disrupts transglycosylation and the overall biosynthesis of the cell wall, ultimately leading to bacterial cell demise. The inhibition of pentaglycine cross-bridging with the stem peptide of glycan chain 2 of the bacterial cell wall, crucial for fortifying cell wall integrity, contributes to this lethal effect [85].

*Rhodococcus equi*, a Gram-positive opportunistic human pathogen, poses a significant threat to immunocompromised individuals, particularly those afflicted with acquired immunodeficiency syndrome (AIDS), with infections being infrequent yet perilous and often culminating in substantial mortality [83,86]. Notably, *Rhodococcus equi* is absent from the rhizobiomes of both plant species examined, whereas two other existing species, namely *R. pyridinivorans* and *R. rhodochrous*, serve as hosts to antibiotic resistance genes (ARGs) prevalent in human populations (Table S11). Conversely, *Streptomyces coelicolor* harbors no human pathogenic species. The presence of approximately 30 *Nocardia* species, including *N. farcinica*, underscores their role as opportunistic pathogens capable of causing various human ailments, spanning pulmonary, cutaneous, or neurological infections [87]. Within this diverse array of *Nocardia* species, both pathogenic and non-pathogenic strains in the rhizospheric bacteria of the two plants, as evidenced in Tables S14 and S15, house a myriad of ARGs, as evidenced by Figure 5, Figure 6 and Figure 7. These observations support the hypothesis of horizontal gene transfer (HGT) events occurring within the rhizospheric soil microbiomes associated with these two plant species.

#### 4.1.4. Antibiotic Target Replacement and Protection

The antibiotic resistance genes (ARGs) *rpoB2* and *RbpA* are integral components of resistance mechanisms involving antibiotic target replacement and protection, respectively. These genes exert their effects on rifampin antibiotics, which target the beta subunit of RNA polymerase (RNAP) in bacteria [88], thereby impeding transcription in Gram-positive bacteria such as *Mycobacterium tuberculosis* and *Rhodococcus* sp. [50,89] (Figure 12). The ARG *rpoB2* orchestrates amino acid substitutions within the beta subunit of RNAP at positions 441 (Asp/Val), 451 (His/Gly), 451 (His/Pro), 454 (Arg/Gln), 456 (Ser/Trp), and/or 456 (Ser/Gln) [51,90]. Meanwhile, the ARG *RbpA* modulates rifampin’s interaction with the σ factor of bacterial RNAP, inducing an allosteric shift in the rifampin binding site within the β subunit [89,91]. Additionally, RbpA protein competes with rifampicin for the β subunit binding site of RNAP, collectively diminishing rifampicin’s affinity for RNAP and impeding its conventional inhibition of the β subunit [50,91]. These changes in β subunit structure disrupt the binding affinity of rifampin to the enzyme, conferring resistance.

### 4.2. Risk of Horizontal Transfer of Soil Rhizospheric ARGs of the Two Wild Plants

In this investigation, we identified 59 bacterial species (Table S11) serving as reservoirs for antibiotic resistance genes (ARGs) commonly observed in humans and coexisting with numerous other species within the same genus in the rhizospheric microbiomes of the two plant species (Tables S14 and S15). These two rhizospheric microbiomes share 51 ARGs with varying resistance mechanisms. It is plausible that closely related genotypes exchange genetic material through processes such as transformation involving mobile genetic elements (MGEs), or through conjugation or transduction. Consequently, there is a potential for pathogenic bacteria, or those acting as repositories for antibiotic resistance genes (ARGs) frequently encountered in human populations, that reside in rhizospheric soil to acquire new antibiotic resistance genes (ARGs) from closely related non-pathogenic species via horizontal gene transfer (HGT). Simultaneously, these non-pathogenic bacteria may come into direct contact with clinical strains in various ecological niches, such as the gut microbiome and through the food chain, facilitating horizontal transfer of ARGs. Consequently, the convergence of original rhizospheric pathogenic bacteria with ARG-transformed clinical strains could engender multi-drug resistant pathogenic bacteria equipped with diverse antibiotic resistance mechanisms, posing an elevated risk to human health.

## 5. Conclusions

In conclusion, this study delineated the shared resistome characteristics and the corresponding resistance mechanisms within the rhizospheric soils of two indigenous plant species endemic to the Mecca region. The potential ramifications of disseminating soil-borne antibiotic resistance genes (ARGs) into the open environment were assessed. The findings offer potential avenues for implementing novel strategies aimed at curtailing the transfer of these ARGs mediated by soil bacteria, which act as reservoirs for antibiotic resistance genes prevalent in human populations, to pathogenic microbes or clinical isolates. Building on recent advances in resistome research, future directions have been proposed to enhance our understanding and management of ARG transmission. Enhanced agricultural and food transportation practices, particularly concerning edible plant components or those employed in traditional medicine, are imperative. Further studies or complementary efforts should focus on developing new, safe, and natural bioactive materials to replace commercial antibiotics. These materials should specifically target these high-risk genes to eliminate ARG within pathogenic microbes and prevent horizontal gene transfer within microbiome communities.

## Data Availability

Supplemental Data can be accessed at https://drive.google.com/drive/folders/1Jn7Lz5zXzh9V26zEDhoG6aXlKE4eejlX?usp=share_link, accessed on 5 May 2024.

## Supplementary Materials

The following supporting information can be downloaded at https://drive.google.com/drive/folders/1Jn7Lz5zXzh9V26zEDhoG6aXlKE4eejlX?usp=share_link. Table S1. Number and description of non-redundant queries of antibiotic resistance genes (ARGs) generated from rhizosphere (R) and bulk (S) soil microbiomes of *Moringa oleifera*. NOVO_MIX refers to reassembled low-copy ORF that is usually found in ≥2 of the six samples, while that preceded by S or R refers to a unique ORF; Table S2. Number and description of non-redundant queries of antibiotic resistance genes (ARGs) generated from rhizosphere (R) and bulk (S) soil microbiomes of *Abutilon fruticosum*. NOVO_MIX refers to reassembled low-copy ORF that is usually found in ≥2 of the six samples, while that preceded by S or R refers to a unique ORF; Table S3. Abundance of antibiotic resistance genes (ARGs) in samples of rhizosphric (R1-R3) and bulk (S1–S3) soil microbiomes of *Moringa oleifera*. These ARGs are ubiquitous in the soil of both wild plants; Table S4. Abundance of antibiotic resistance genes (ARGs) in samples of rhizosphric (R1-R3) and bulk (S1–S3) soil microbiomes of *Abutilon fruticosum*. These ARGs are ubiquitous in the soil of both wild plants; Table S5. Number of non-redundant queries (≥3) concerning antibiotic resistance genes (ARGs), inclusive of their resistance mechanisms and bacterial hosts, prevalent within rhizospheric (S) soil microbiomes of the two indigenous species *Moringa oleifera* (MO) and *Abutilon fruticosum* (AF). The asterisk denotes ARGs exhibiting multiple resistance mechanisms; Table S6. Data extracted from CARD (https://card.mcmaster.ca, accessed on 5 May 2024) to elucidate prevalent antibiotic resistance genes (ARGs) involved in the antibiotic efflux resistance mechanism within rhizospheric and bulk soil microbiomes of *Moringa oleifera* and *Abutilon fruticosum*. Antibiotic resistance via this mechanism entails the extrusion of antibiotics from the cell [66]; Table S7. Data extracted from CARD (https://card.mcmaster.ca) to elucidate prevalent antibiotic resistance genes (ARGs) involved in the antibiotic inactivation resistance mechanism within rhizospheric and bulk soil microbiomes of *Moringa oleifera* and *Abutilon fruticosum*. Antibiotic resistance via this mechanism entails the enzymatic inactivation of antibiotics to confer drug resistance [73]; Table S8. Data extracted from CARD site (https://card.mcmaster.ca) pertaining to antibiotic resistance genes (ARGs) involved in the antibiotic target alteration resistance mechanism within rhizospheric and bulk soil microbiomes of *Moringa oleifera* and *Abutilon fruticosum*. Antibiotic resistance conferred by this mechanism arises from mutational alteration or enzymatic modification of antibiotic targets, leading to resistance against antibiotics; Table S9. Data extracted from CARD site (https://card.mcmaster.ca) concerning antibiotic resistance genes (ARGs) engaged in the antibiotic target protection resistance mechanism within rhizospheric soil microbiomes of *Moringa oleifera* and *Abutilon fruticosum*. Antibiotic resistance facilitated by this mechanism arises from genes encoding bacterial RNA polymerase-binding protein family, functioning as transcription factors that bind to the sigma subunit of RNA polymerase [89,91]; Table S10. Data extracted from CARD site (https://card.mcmaster.ca) regarding antibiotic resistance genes (ARGs) involved in the antibiotic target replacement resistance mechanism within rhizospheric soil microbiomes of microbiomes of *Moringa oleifera* and *Abutilon fruticosuum*. Antibiotic resistance stemming from this mechanism arises through the replacement or substitution of antibiotic targets, leading to resistance against antibiotics [60]; Table S11. Bacterial inhabitants hosting antibiotic resistance genes (ARGs) prevalent in humans. The dataset was generated through the utilization of the Resistance Gene Identifier (RGI), a computational tool designed for the detection of potential antimicrobial resistance genes within submitted sequence annotation data sourced from the Comprehensive Antibiotic Resistance Database (CARD). This analysis adhered to the Technical Rules for Biological Agents (TRBA) stipulated by the German Federal Institute for Occupational Safety and Health (TRBA 466), as referenced by Nelkner et al. (2019). Species highlighted in bold (59) signify bacteria observed within the rhizospheric soil of the two indigenous plant species; Table S12. Abundance of non-redundant genes at the genus-species level within metagenomes obtained from rhizospheric (R) soils of *Moringa oleifera* in three replicates; Table S13. Abundance of non-redundant genes at the genus-species level within metagenomes obtained from rhizospheric (R) soil of *Abutilon fruticosum* in three replicates; Table S14. Abundance of non-redundant gene queries of the bacteria harboring ARGs at the genus/species level that are common in rhizospheric (S) soil microbiomes of the wild species *Moringa oleifera*. Bacteria highlighted in bold denote microbial inhabitants within the rhizospheric soil of the two indigenous plant species known to serve as reservoirs for antibiotic resistance genes (ARGs) commonly observed in human. Complete list of these bacteria is shown in Table S11; Table S15. Abundance of non-redundant gene queries of the bacteria harboring ARGs at the genus/species level that are common in rhizospheric (S) soil microbiomes of the wild species *Abutilon fruticosum*. Bacteria highlighted in bold denote microbial inhabitants within the rhizospheric soil of the two indigenous plant species known to serve as reservoirs for antibiotic resistance genes (ARGs) commonly observed in human. Complete list of these bacteria is shown in Table S11.
